# Efficacy and safety of ciprofol versus propofol for anesthesia induction in adult patients received elective surgeries: a meta‑analysis

**DOI:** 10.1186/s12871-024-02479-9

**Published:** 2024-03-07

**Authors:** Dilireba Ainiwaer, Wanwei Jiang

**Affiliations:** Department of anesthesiology, Affiliated Zhongshan Hospital of Dalian University, Dalian, 116001 China

**Keywords:** Elective surgery, General anesthesia, Ciprofol, Propofol, Meta-analysis

## Abstract

**Background:**

Propofol is use widely used in anesthesia, known for its effectiveness, may lead to cardiopulmonary issues in some patients. Ciprofol has emerged as a possible alternative to propofol because it can achieve comparable effects to propofol while causing fewer adverse events at lower doses. However, no definitive conclusion has been reached yet. This meta-analysis aimed to evaluate the efficacy and safety of ciprofol versus propofol in adult patients undergoing elective surgeries under general anesthesia.

**Methods:**

We searched PubMed, EMBASE, the Cochrane library, Web of Science, and Chinese National Knowledge Infrastructure (CNKI) to identify potentially eligible randomized controlled trials (RCT) comparing ciprofol with propofol in general anesthesia until September 30, 2023. The efficacy outcomes encompassed induction success rate, time to onset of successful induction, time to disappearance of eyelash reflex, and overall estimate means in Bispectral Index (BIS). Safety outcomes were assessed through time to full alertness, incidence of hypotension, incidence of arrhythmia, and incidence of injection-site pain. Continuous variables were expressed as mean difference (MD) with 95% confidence interval (CI), and dichotomous variables were expressed as risk ratio (RR) with 95% CI. Statistical analyses were performed using RevMan 5.4 and STATA 14.0. The quality of the evidence was rated through the grading of recommendations, assessment, development and evaluation (GRADE) system.

**Results:**

A total of 712 patients from 6 RCTs were analyzed. Meta-analysis suggested that ciprofol was equivalent to propofol in terms of successful induction rate, time to onset of successful induction, time to disappearance of eyelash reflex, time to full alertness, and incidence of arrhythmia, while ciprofol was better than propofol in overall estimated mean in BIS (MD: -3.79, 95% CI: -4.57 to -3.01, *p* < 0.001), incidence of hypotension (RR: 0.63, 95% CI: 0.42 to 0.94, *p* = 0.02), and incidence of injection-site pain (RR: 0.26, 95% CI: 0.14 to 0.47, *p* < 0.001). All results were supported by moderate to high evidence.

**Conclusions:**

Ciprofol may be a promising alternative to propofol because it facilitates achieving a satisfactory anesthesia depth and results in fewer hypotension and injection-site pain. However, we still recommend conducting more studies with large-scale studies to validate our findings because only limited data were accumulated in this study.

**Trial registration:**

PROSPERO 2023 CRD42023479767.

**Supplementary Information:**

The online version contains supplementary material available at 10.1186/s12871-024-02479-9.

## Background

Propofol is a widely used anesthetic drug, with common applications including conscious sedation [1, 2] and general anesthesia [3, 4]. The efficacy and safety of the use of propofol for conscious sedation and general anesthesia have been supported by solid evidence [5–7]; however, studies have shown that propofol can also produce various side effects, such as injection-site pain [8], propofol-related infusion syndrome [9] and an increased risk of infection [10]. As a result, it is imperative to develop a novel anesthetic drug that is as effective as propofol but has fewer side effects [11].

Ciprofol (HSK3486) is a newly developed highly selective γ-aminobutyric acid (GABA) receptor agonist [12], which has become a new type of intravenous sedative anesthetic drug with desirable properties, such as rapid onset of action, fast recovery, minimal pain on injection, and stable cardiopulmonary function [13–15]. Clinical studies have shown that 0.4 to 0.5 mg/kg ciprofol is equivalent to 2.0 mg/kg propofol in sedative and anesthetic profile during colonoscopy [16, 17]. All these advantages make ciprofol a promising alternative to propofol in conscious sedation and general anesthesia [15, 17].

Up to date, several randomized controlled trials (RCTs) [18–23] have investigated the efficacy and safety of ciprofol compared with propofol in patients undergoing elective surgeries under general anesthesia, but reported conflicting results. Some studies have shown no statistical difference between ciprofol and propofol in the time to onset of successful induction [18, 19, 22, 23] and in the incidence of hypotension [19–21, 23], whereas other studies reported conflicting results in terms of the time to onset of successful induction [20, 21] and incidence of hypotension [18, 22]. Furthermore, it is important to note that all these studies included only limited sample size, thus inevitably increasing the risk of producing misleading outcomes.

Therefore, the purpose of this meta-analysis was to systematically evaluate the comparative anesthetic efficacy and safety of ciprofol versus propofol in patients undergoing elective surgeries under general anesthesia, with a view to providing evidence for informing the selection of the optimal anesthetic drug.

## Methods

We strictly followed the Cochrane handbook to conduct this meta-analysis [24]. The Preferred Reporting Items for Systematic Reviews and Meta-Analysis (PRISMA) 2020 statement was cited as the guidance for reporting this meta-analysis [25]. Institutional review approval and informed consent were not required because we collected data directly from previously published studies.

### Selection criteria

Studies were eligible if (a) adult patients underwent non-emergency, non-cardiothoracic, and non-neurological elective surgeries under general anesthesia, with American Society of Anesthesiologists (ASA) status I/II; (b) anesthesia induction in the study group was performed using ciprofol (0.4–0.5 mg/kg); (c) anesthesia induction in the control group was performed using propofol (2.0 mg/kg); (d) they reported at least one of the following outcomes, including induction success rate, onset of successful induction, time to disappearance of eyelash reflex, time to fully alertness, overall estimated mean in bispectral index (BIS), and incidence of hypotension, arrhythmia and injection-site pain; and (e) only randomized controlled trials (RCTs) were considered to meet inclusion criteria.

Studies were excluded if they (a) used ineligible study designs, such as animal study, single-arm trial, case report, and review; (b) conference abstract without essential data for statistical analysis; (c) evaluated the synthetic effect of ciprofol combined with propofol rather than effect of individual anesthetic drug; (d) repeated report of the same population.

### Search strategy

A systematic search was conducted in PubMed, EMBASE, the Cochrane library, Web of Science and Chinese National Knowledge Infrastructure (CNKI) to retrieve potentially eligible studies that compared ciprofol with propofol in adult patients underwent elective surgery under general anesthesia. The latest date to update search was September 30, 2023. We used “ciprofol,” “propofol,” and “random”, as well as their analogs as search terms, and the strategy of combining full text and medical subject heading (MeSH) was adopted as the principle for constructing search strategy. Supplementary Table 1 summarized the detailed search strategies of all target databases. Additional studies were also retrieved using manual search of the reference lists of eligible studies and reviews that investigated the same topic.

### Selection processes

Two authors (Wanwei Jiang and Dilireba Ainiwaer) independently performed study selection following three steps. First, we removed duplicate studies using EndNote software. Second, we excluded irrelevant studies based on title and abstract screening. Third, we identified studies that met our selection criteria based on full-text screening. Consensus was employed to resolve disagreements between the two authors.

### Data collection

Data were collected independently by two authors using a pre-designed standard data extraction form based on MS Excel 2022 (Microsoft Corporation, the USA). Specifically, we collected the following data from all eligible studies: study characteristics (the first author’s name, country, year of publication, surgical procedure, protocol of administration of ciprofol and propofol, general anesthesia protocol, and muscle relaxant), patient characteristics (sample size, the number of female patients, average age, body mass index [BMI], ASA status, and operative duration), outcomes data, and information for methodological quality.

### Outcome definition

Induction success rate was defined as the percentage of successful induction cases in each group, with successful induction defined as not requiring any alternative sedative or anesthetic drug or requiring > 2 top-up study drug doses after the start of study drug administration. The time to onset of successful induction refers to the time from the initiation of study drug treatment until the patient achieved a Modified Observer’s Assessment of Alertness/Sedation (MOAA/S) score of ≤ 1. The time to disappearance of eyelash reflex was defined as the time before the eyelash reflex is completely lost. The overall estimated mean in BIS was the difference between the two groups in the overall estimated value in BIS after achieving sedation. The time to full alertness refers to the time from drug withdrawal to extubation (MOAA/S of 5 for three consecutive assessments). The definition of hypotension was left to each study [26]. Arrhythmia was the composite of bradycardia and tachycardia in this meta-analysis. Bradycardia and tachycardia refer to a heart rate < 50 beats/min and heart rate > 100 beats/min with a duration of > 30s, respectively. Injection site pain as detected by a withdrawal response or a numeric rating scale value of ≥ 3.

### Risk of bias assessment

The risk of bias for each eligible study was independently assessed by two authors (Wanwei Jiang and Dilireba Ainiwaer) using the revised Cochrane risk of bias (RoB) tool [27]. The tool was designed with 5 domains including randomization process, deviations from the intended interventions, missing outcome data, measurement of the outcome, and selection of the reported result. Each domain would be rated as ‘low risk,’ ‘some concerns,’ or ‘high risk’ based on the actual information provided by each study. The overall risk of an individual study was rated as low if 5 domains were marked as low risk, and if one or more domains were rated as high risk, the overall risk was rated as high. In addition, the overall risk of an individual study was rated as having some concerns if there was one or more domains of some concerns but no domain of high risk.

### Statistical analysis

The estimates for continuous variables were summarized with mean difference (MD) with 95% confidence interval (CI), and estimates for dichotomous variables were expressed as risk ratio (RR) with 95% CI [24]. We assessed statistical heterogeneity between studies by using the Cochrane Q statistic and I^2^ statistic [28]. Statistically significant heterogeneity was considered if *p* < 0.1 and I^2^ ≥ 50%, and the random-effects model was used for meta-analysis [29]. In contrast, statistical heterogeneity was considered as low if *p* ≥ 0.1 and I^2^ < 50%, and the fixed-effects model was selected for meta-analysis. We also employed the leave-one-out method to conduct sensitivity analysis. Although the number of included studies was less than ten [30], we still used both funnel plot and Egger’s test to assess publication bias. Review Manager (RevMan) version 5.4 (the Nordic Cochrane Centre, the Cochrane Collaboration, Copenhagen, Denmark) and STATA 14.0 (StataCorp LP, College Station, USA) [31] were used for all statistical analyses.

### The quality of evidence

We used the grading of recommendations, assessment, development and evaluation (GRADE) system to assess the quality of the evidence [32]. Using the GRADE method, the level of evidence for each outcome would be rated as ‘high,’ ‘moderate,’ ‘low,’ or ‘very low’. According to the GRADE method, the initial level of evidence for RCT is the highest level; however, the level of evidence would be downgraded based on limitations in the 5 aspects: risk of bias, consistency, indirectness, imprecision, and publication bias.

## Results

### Study retrieval

**Fig. 1:**
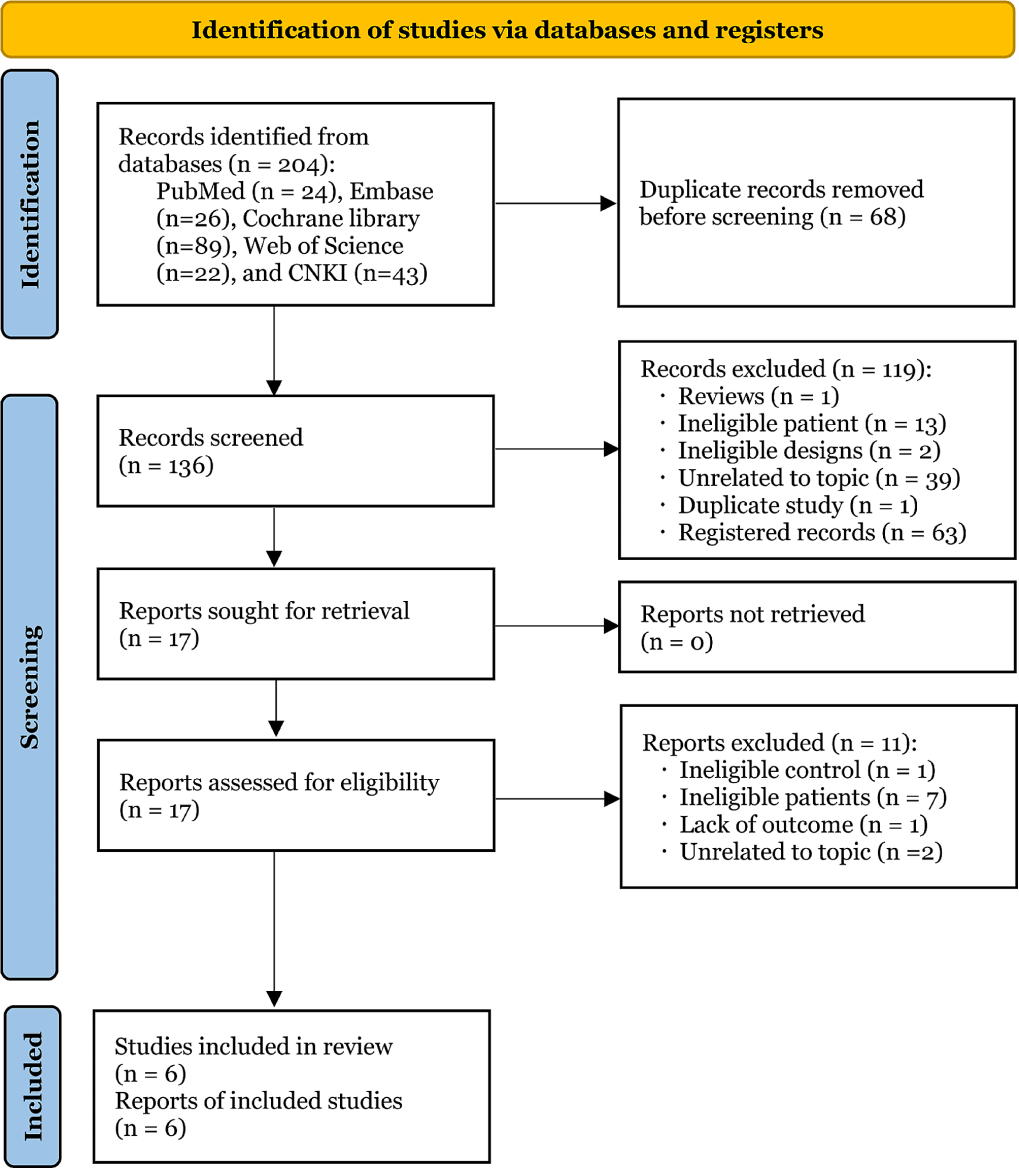
PRISMA flow diagram of study screening and selection

We retrieved a total of 204 potentially eligible studies from five electronic databases, including PubMed (*n* = 24), EMBASE (*n* = 26), Cochrane library (*n* = 89), Web of Science (*n* = 22), and CNKI (*n* = 43). After excluding 68 duplicate studies and 119 irrelevant studies, 17 studies were retained for final eligibility assessment. After exclusion of 11 ineligible studies due to ineligible control (*n* = 1), ineligible patients (*n* = 7), lack of outcome (*n* = 1), and unrelated to topic (*n* = 2), 6 eligible RCTs [18–23] were eventually included for data analysis. The detailed process of study screening is depicted in Fig. 1.

### Study characteristics

Table 1 summarizes the detailed basic characteristics of eligible studies. All eligible studies [18–23] were conducted in China between 2022 and 2023. Three studies [18, 21, 23] recruited patients undergoing elective surgeries under general anesthesia, one study [19] recruited patient undergoing non-emergency, non-cardiothoracic, and non-brain elective surgeries, and other two studies [20, 22] recruited patients who were assigned to receive gynecological ambulatory surgeries. The sample size of individual studies ranged from 40 to 176, with a cumulative total of 712 cases. Five studies [18, 19, 21–23] used 0.6 mg/kg Rocuronium as a muscle relaxant, while the other one study [20] used 0.2 mg/kg Mivacurium as a muscle relaxant.

**Table 1:** Basic characteristics of eligible studies (*n* = 6)

Study	Study design	Characteristics of patients					Details of intervention and control				Outcomes
		Surgical procedure	No.	Females (%)	Age, years	BMI, kg/m^2^	Doses	Protocol of administration	General anesthesia protocol	Muscle relaxant	
Chen et al., 2022	RCT	Elective gynecological surgery	60	60 (100.0%)	33.9 ± 9.1	22.2 ± 3.2	Ciprofol 0.4 mg/kg	Manual injection within 30s	Intravenous midazolam (0.03 mg/kg) and sufentanil (0.3 g/kg)	Rocuronium (0.6 mg/kg)	SAI, TOSI, TDER, BIS, HT, AT, IP
			60	60 (100.0%)	33.8 ± 9.6	21.4 ± 2.8	Propofol 2 mg/kg				
Liang et al., 2023	RCT	Non-emergency, non-cardiothoracic, and non-brain elective surgeries	86	63 (73.3%)	38.5 ± 10.1	23.3 ± 2.8	Ciprofol 0.4 mg/kg	Manual injection within 30s	Intravenous midazolam (0.03 mg/kg) and sufentanil (0.3 g/kg)	Rocuronium (0.6 mg/kg)	SAI, TOSI, BIS, TFA, HT, AT, IP
			42	32 (76.2%)	40.5 ± 10.1	23.3 ± 3.0	Propofol 2 mg/kg				
Wang et al., 2022	RCT	Elective surgeries under general anesthesia	88	56 (63.6%)	38.5 ± 12.1	23.3 ± 2.9	Ciprofol 0.4 mg/kg	Manual injection within 30s	Intravenous midazolam (0.03 mg/kg) and sufentanil (0.3 g/kg)	Rocuronium (0.6 mg/kg)	SAI, TOSI, TDER, BIS, HT, AT, IP
			88	57 (65.0%)	41.1 ± 11.1	23.3 ± 3.1	Propofol 2 mg/kg				
Zeng, et al., 2022	RCT	Elective surgeries under general anesthesia	30	19 (63.3%)	42.5 ± 10.3	23.7 ± 3.0	Ciprofol 0.4 mg/kg	Manual injection within 30s	Intravenous midazolam (0.03 mg/kg) and sufentanil (0.3 g/kg)	Rocuronium (0.6 mg/kg)	SAI, TOSI, BIS, TFA, HT, AT, IP
			10	7 (70.0%)	46.4 ± 11.2	23.6 ± 3.6	Propofol 2 mg/kg				
Yin et al., 2023	RCT	Gynaecological ambulatory surgery under general anesthesia	60	60 (100.0%)	33.9 ± 9.1	22.4 ± 3.4	Ciprofol 0.4 mg/kg	Manual injection within 30s	Intravenous midazolam (0.03 mg/kg) and sufentanil (0.3 g/kg)	Rocuronium (0.6 mg/kg)	SAI, TOSI, TDER, BIS, HT, AT, IP
			60	60 (100.0%)	33.8 ± 9.6	21.5 ± 3.4	Propofol 2 mg/kg				
Man et al., 2023	RCT	Gynaecological ambulatory surgery under general anesthesia	64	64 (100.0%)	42.2 ± 9.5	22.8 ± 2.2	Ciprofol 0.4 mg/kg	Pump injection for 60s	Intravenous alfentanil (20 µg/kg)	Mivacurium (0.2 mg/kg)	SAI, TOSI, BIS, TFA, HT, AT, IP
			64	64 (100.0%)	44.1 ± 9.4	23.3 ± 2.6	Propofol 2 mg/kg				

RCT, randomized controlled trial; BMI, body mass index; SAI, successful anesthesia induction; TOSI, time to onset of successful induction; TDER, time to disappearance of eyelash; BIS, bispectral index; TFA, time to fully alertness; HT, hypotension; AT, arrhythmia; IP, injection-site pain

### Risk of bias assessment

**Fig. 2:**
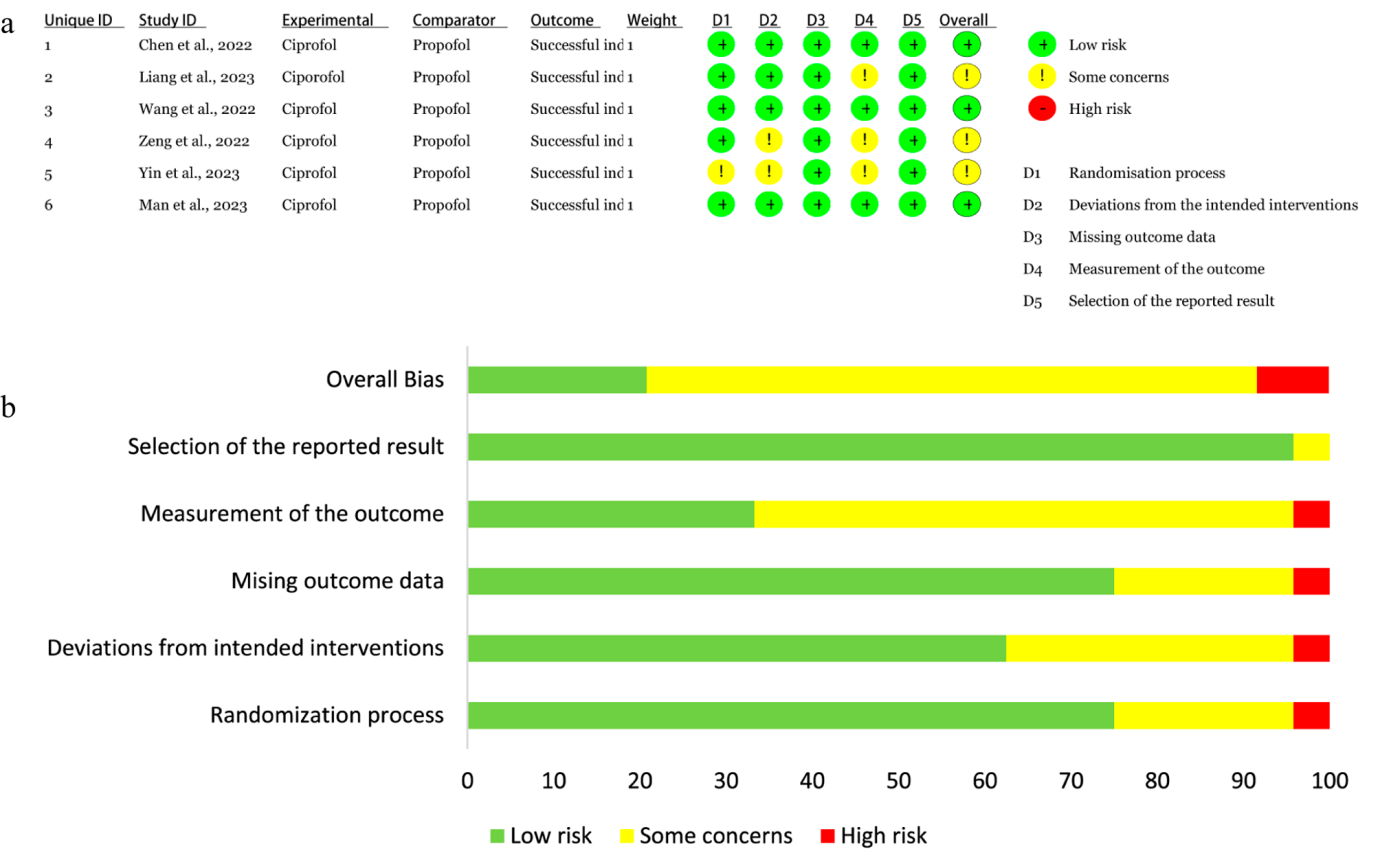
Risk of bias summary **(a)** and graph **(b)**

Five studies [18–21, 23] were rated as low risk in randomization process, but one study [22] was rated as having some concerns. Two studies [22, 23] were rated as having some concerns regarding deviations from the intended interventions, whereas the other four studies [18–21] were rated as low risk in this domain. Three studies [19, 22, 23] were rated as having low risk in terms of measurement of the outcome, and the other three studies [18, 20, 21] were rated as having some concerns in this domain. All studies [18–23] were rated as having low risk in missing outcome data and selection of the reported result. Finally, three studies [18, 20, 21] were rated as having a low risk of overall bias, while the other studies [19, 22, 23] were rated as having some concerns for overall bias. Detailed results of the risk of bias assessment are showed in Fig. 2.

### Meta-analysis of efficacy

#### Induction success rate

**Fig. 3:**
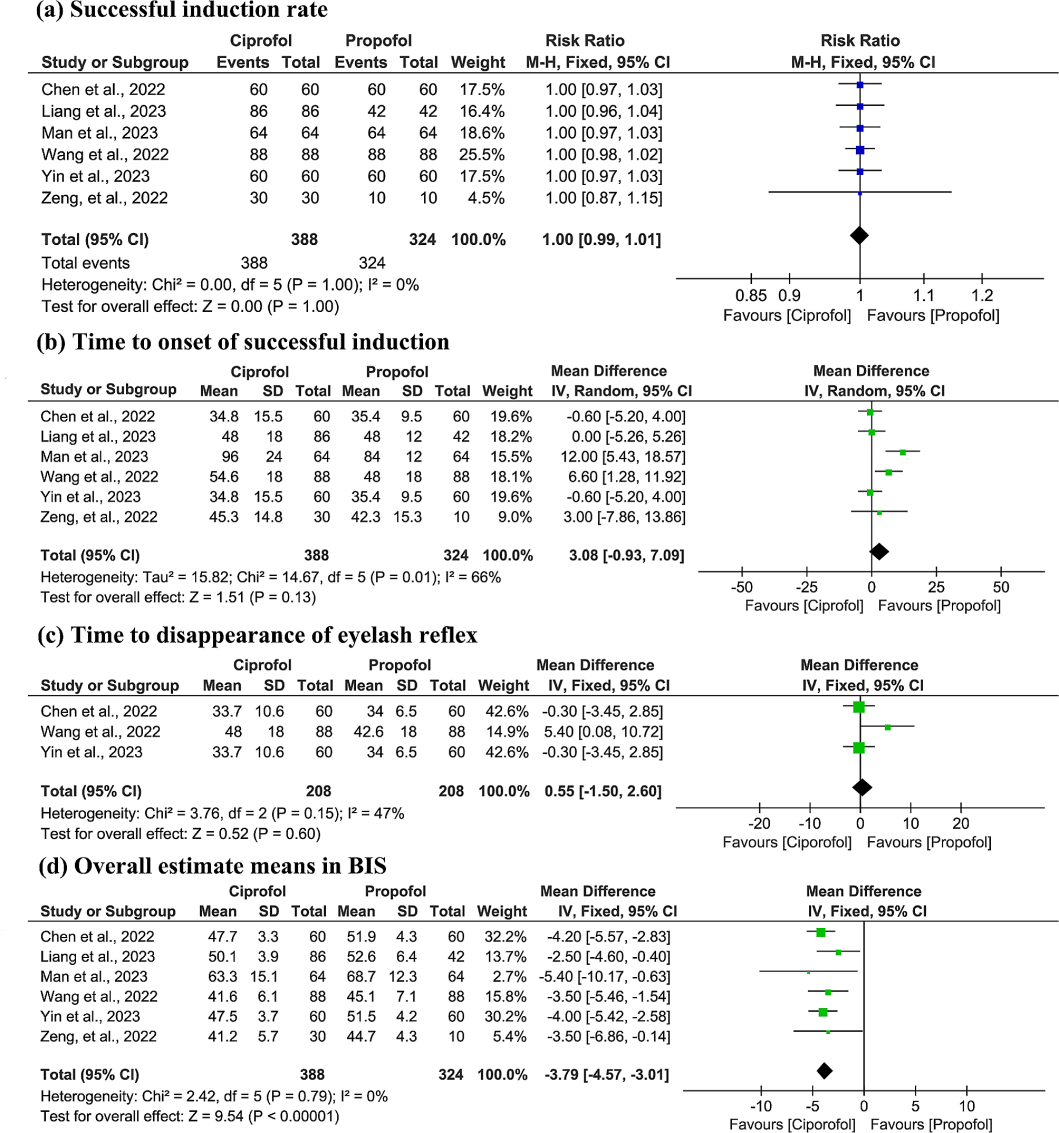
Forest plots of successful induction rate **(a)**, time to onset of successful induction **(b)**, time to disappearance of eyelash reflex **(c)**, and overall estimated mean in BIS **(d)** between ciprofol and propofol groups

All eligible studies [18–23] evaluated successful induction rate, which were 100% in both groups. As shown in Fig. 3a, no significant statistical heterogeneity was detected (*p* = 1.00, I^2^ = 0.0%), therefore the fixed-effects model was used for meta-analysis. The merged result showed that both ciprofol and propofol achieved the same successful induction rate (RR: 1.00, 95% CI: 0.99 to 1.01, z = 0.00, *p* = 1.00), which was supported by moderate evidence (Table 2).

**Table 2:** GRADE evidence profile of all outcomes

No of studies	Study design	Certainty assessment					№ of patients		Effect		Certainty	Importance
		RoB	Inconsistency	Indirectness	Imprecision	Other considerations	Ciprofol	Propofol	Relative (95% CI)	Absolute (95% CI)		
**Successful induction rate**												
6	RCT	not serious	not serious	not serious	not serious	publication bias strongly suspected^a^	388/388 (100.0%)	324/324 (100.0%)	**RR 1.00** (0.99 to 1.01)	**0 fewer per 1,000** (from 10 fewer to 10 more)	⨁⨁⨁◯ Moderate	Critical
**Time to onset of successful induction**												
6	RCT	not serious	serious^b^	not serious	not serious	none	388	324	-	MD **3.08 s higher** (0.93 lower to 7.09 higher)	⨁⨁⨁◯ Moderate	Critical
**Time to disappearance of eyelash reflex**												
3	RCT	not serious	serious^c^	not serious	not serious	none	208	208	-	MD **0.55 s higher** (1.5 lower to 2.6 higher)	⨁⨁⨁◯ Moderate	Critical
**Overall estimated mean in BIS**												
6	RCT	not serious	not serious	not serious	not serious	none	388	324	-	MD **3.79 lower** (4.57 lower to 3.01 lower)	⨁⨁⨁⨁ High	Critical
**Time to full alertness**												
3	RCT	not serious	not serious	not serious	not serious	none	180	116	-	MD **0.67 min higher** (0.03 lower to 1.36 higher)	⨁⨁⨁⨁ High	Critical
**Incidence of hypotension**												
6	RCT	not serious	serious^b^	not serious	not serious	none	106/388 (27.3%)	127/324 (39.2%)	**RR 0.63** (0.42 to 0.94)	**145 fewer per 1,000** (from 227 fewer to 24 fewer)	⨁⨁⨁◯ Moderate	Important
**Incidence of arrhythmia**												
6	RCT	not serious	not serious	not serious	not serious	none	44/388 (11.3%)	41/324 (12.7%)	**RR 0.81** (0.55 to 1.21)	**24 fewer per 1,000** (from 57 fewer to 27 more)	⨁⨁⨁⨁ High	Important
**Incidence of injection-site pain**												
6	RCT	not serious	serious^a^	not serious	not serious	none	34/388 (8.8%)	146/324 (45.1%)	**RR 0.26** (0.14 to 0.47)	**333 fewer per 1,000** (from 388 fewer to 239 fewer)	⨁⨁⨁◯ Moderate	Important

**RoB**, risk of bias; **CI**, confidence interval; **MD**, mean difference; **RR**, risk ratio

**Explanations**

a. Egger’s test shows the risk of publication bias

b. An inconsistency factor of 66% was estimated

c. An inconsistency factor of 47% was estimated

#### Time to onset of successful induction

All studies [18–23] evaluated the time to onset of successful induction between ciprofol and propofol groups. As shown in Fig. 3b, significant statistical heterogeneity was detected (*p* = 0.01, I^2^ = 66.0%), therefore the random-effects model was used for meta-analysis. The merged result showed that there was no significant difference between ciprofol and propofol in this outcome (MD: 3.08, 95% CI: -0.93 to 7.09, z = 1.51, *p* = 0.13), which was supported by the moderate evidence (Table 2).

#### Time to disappearance of eyelash reflex

Three studies [18, 21, 22] evaluated the time to disappearance of eyelash reflex between ciprofol and propofol groups. As shown in Fig. 3c, no statistical heterogeneity was detected (*p* = 0.15, I^2^ = 47.0%), therefore the fixed-effects model was used for meta-analysis. The merged result showed that ciprofol was comparable to propofol in this outcome (MD: 0.55, 95% CI: -1.50 to 2.60, z = 0.52, *p* = 0.60), which was supported by the moderate evidence (Table 2).

#### Overall estimate means in BIS

All studies [18–23] evaluated the overall estimated means in BIS between ciprofol and propofol. As shown in Fig. 3d, statistical heterogeneity was insignificant (*p* = 0.79, I^2^ = 0.0%), therefore the fixed-effects model was used for meta-analysis. The merged result showed that ciprofol was better than propofol in terms of overall estimated means in BIS (MD: -3.79, 95% CI: -4.57 to -3.01, z = 9.54, *p* < 0.001), which was supported by the high evidence (Table 2).

### Meta-analysis of safety

#### Time to full alertness

**Fig. 4:**
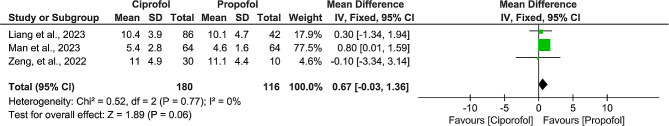
Forest plot of time to full alertness between ciprofol and propofol groups

Three studies [19, 20, 23] evaluated to time to full alertness between ciprofol and propofol. As shown in Fig. 4, no significant statistical heterogeneity was detected (*p* = 0.77, I^2^ = 0.0%), therefore the fixed-effects model was used for meta-analysis. The merged result showed that there was no difference in this outcome between ciprofol and propofol groups (MD: 0.67, 95% CI: -0.03 to 1.36, z = 1.89, *p* = 0.06), which was supported by the high evidence (Table 2).

#### Incidence of hypotension

**Fig. 5:**
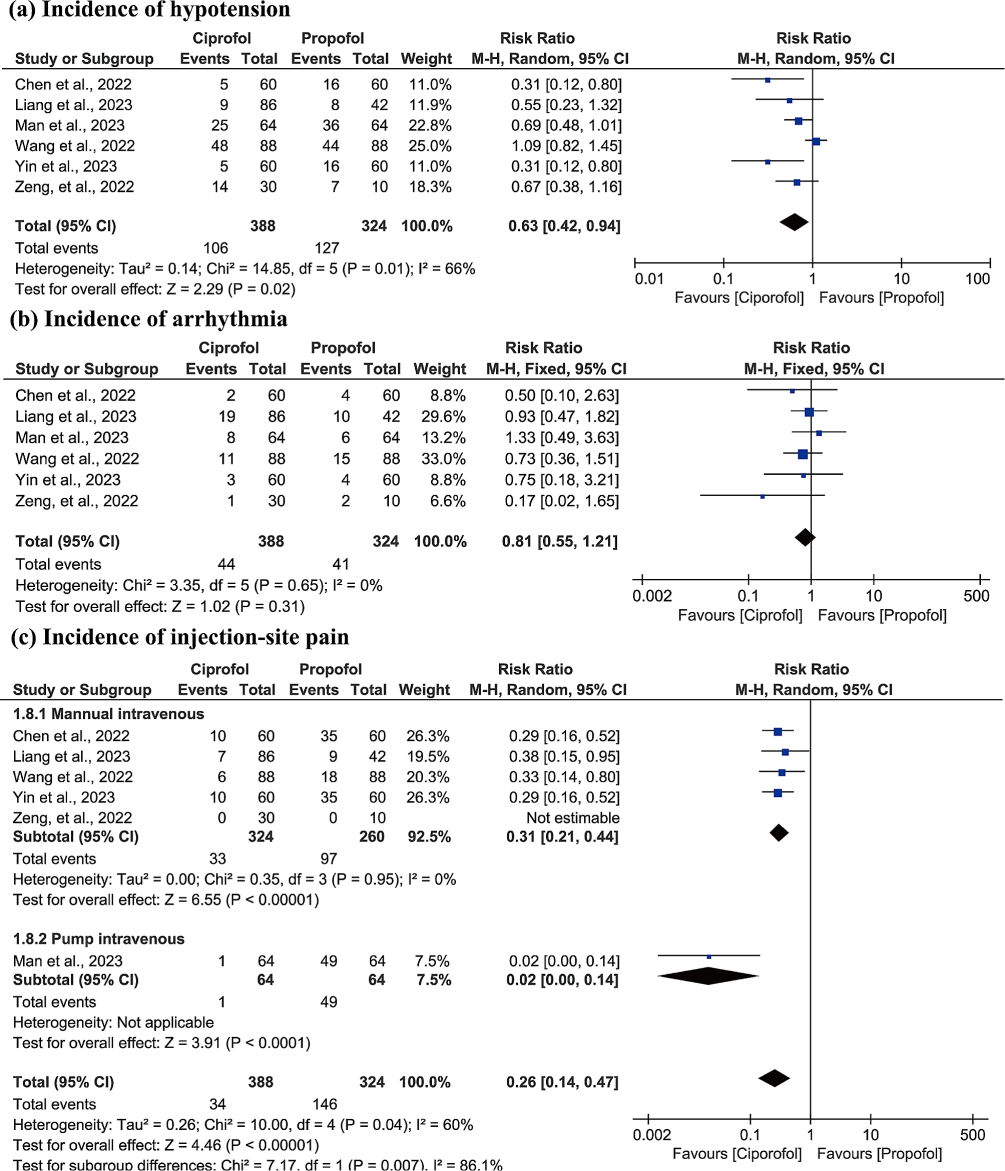
Forest plots of time to incidence of hypotension **(a)**, incidence of arrhythmia **(b)**, and incidence of injection-site pain **(c)** between ciprofol and propofol groups

All studies [18–23] evaluated the incidence of hypotension between ciprofol and propofol. As shown in Fig. 5a, significant statistical heterogeneity was detected (*p* = 0.01, I^2^ = 66.0%), therefore the random-effects model was used for meta-analysis. The merged result showed that, compared with propofol, ciprofol was associated with lower incidence of hypotension (RR: 0.63, 95% CI: 0.42 to 0.94, z = 2.29, *p* = 0.02), which was supported by the moderate evidence (Table 2).

#### Incidence of arrhythmia

All studies [18–23] evaluated the incidence of arrhythmia between ciprofol and propofol. As shown in Fig. 5b, no significant statistical heterogeneity was detected (*p* = 0.65, I^2^ = 0.0%), therefore the fixed-effects model was used for meta-analysis. The merged result showed that there was no statistical difference in the incidence of arrhythmia between ciprofol and propofol (RR: 0.81, 95% CI: 0.55 to 1.21, z = 1.02, *p* = 0.31), which was supported by very the high evidence (Table 2).

#### Incidence of injection-site pain

All studies [18–23] evaluated the incidence of injection-site pain between ciprofol and propofol, but one study [23] was excluded from data analysis because it reported zero event in the both groups. As shown in Fig. 5c, significant statistical heterogeneity was detected (*p* = 0.04, I^2^ = 60.0%), therefore the random-effects model was used for meta-analysis. In addition, because previous studies have demonstrated the correlation between injection speed and the incidence of injection-site pain, therefore subgroup analysis was also introduced according to the method of injection (manual intravenous vs. pump intravenous). The merged result showed that, compared with propofol, ciprofol was associated with significantly lower incidence of injection-site pain (RR: 0.26, 95% CI: 0.14 to 0.47, z = 4.46, *p* < 0.001), which was supported by the moderate evidence (Table 2). Subgroup analysis showed that patients who received ciprofol with manual intravenous (RR: 0.31, 95% CI: 0.21 to 0.44, z = 6.55, *p* < 0.001) or pump intravenous (RR: 0.02, 95% CI: 0.00 to 0.14, z = 3.91, *p* < 0.001) experience significantly lower incidence of injection-site pain, while pump intravenous might be better than manual intravenous (*p* = 0.007, I^2^ = 86.1%).

### Sensitivity analysis

Detailed results of the sensitivity analysis are shown in supplementary Fig. 1 to 3. The results showed that the merged results of individual meta-analyses did not change significantly after excluding one study a time, meaning that all pooled results were robust.

### Publication bias

Funnel plots of all outcomes are displayed in supplementary Fig. 4 to 6. Visual inspection for these funnel plots showed symmetric outlines; however, the results of Egger’s test showed that the successful induction rate (*p* = 0.024) and incidence of hypotension (*p* = 0.012) were at risk of publication bias. For the other 6 outcomes, Egger’s test showed evidence supporting the absence of publication bias, with p-values of 0.372, 0.602, 0.615, 0.692, 0.184, and 0.162 for the time to onset of successful induction, the time to disappearance of eyelash reflex, time to full alertness, overall estimated mean in BIS, incidence of arrhythmia, and incidence of Injection-site pain, respectively.

## Discussion

Ciprofol has recently emerged as a potential alternative to propofol due to its better GABAA receptor affinity. However, no definitive conclusion has been drawn as to whether ciprofol is better than propofol in patients undergoing elective surgeries under general anesthesia. In this meta-analysis, we accumulated a total of 712 patients to further evaluate the comparative efficacy and safety of ciprofol versus propofol in patients underwent elective surgeries under general anesthesia. This present meta-analysis indicated that ciprofol was more effective in providing deeper anesthesia (as shown by overall higher estimated mean in BIS) and led to lower incidences of hypotension and injection-site pain compared with propofol. When compared to propofol, ciprofol had a similar rate of induction rate, time to onset of successful induction, time to disappearance of eyelash reflex, time to full alertness, and incidence of arrhythmia. As a newly developed intravenous anesthetic drug, ciprofol exhibits good pharmacodynamic properties, including rapid onset of action and rapid recovery [14]. Furthermore, it binds more tightly to the GABA type A (GABAA) receptor than propofol and exhibits lower lipophilicity and a more appropriate steric bulk [18]. Therefore, ciprofol has been regarded as a promising alternative to propofol [16]. In this meta-analysis, we found that ciprofol was comparable to propofol in terms of successful induction rate, time to onset of successful induction, time to disappearance of eyelash reflex, time to full alertness, and incidence of arrhythmias. These results provide evidence that ciprofol has similar sedative and anesthetic efficacy to propofol in general anesthesia.

Intraoperative accidental awareness is a very serious consequence of general anesthesia that can cause patients to experience recurring anxiety, nightmares and psychological repercussion, and can also lead to posttraumatic stress disorder in more severe cases [33]. Anesthesia depth is one of the major contributors to the occurrence of intraoperative accidental awareness [34]. So, an appropriate anesthesia depth should be achieved and maintained during intraoperative maintenance. BIS is an electroencephalogram-derived parameter used to monitor the depth of anesthesia during operation [35], with BIS < 60 indicating sedation status [19]. However, deep anesthesia (BIS < 40) must also be avoided as it has been found to be associated with increased risk of electroencephalogram burst suppression and cardiovascular dysfunctions [36]. This meta-analysis showed that the overall estimated BIS in patients received ciprofol was less than that in patients received propofol, suggesting that ciprofol achieved a better anesthesia depth than propofol.

Hypotension is also one of the known common adverse effect of the administration of propofol for general anesthesia [20]. Growing evidence suggests that intraoperative hypotension is linked to increased rates of damage to vital organs (e.g., heart, kidneys and brain) and mortality in high-risk patients [37–39]. In this meta-analysis, we found that the administration of ciprofol was associated with a significantly lower incidence of hypotension than propofol, which was consistent with the results of some previous studies [19–21].

Injection pain is among the most frequently reported propofol-related adverse effects, with an estimated incidence of 50–80% [40–42]. This meta-analysis showed an accumulated incidence of 45.1% for pain on injection, while the incidence of injection-site pain was only 8.8% in ciprofol group. Many factors may contribute to the occurrence of injection-site pain, such as the concentration of the drug and injection speed. Ciprofol is an isomer of propofol, and a cyclopropyl group is inserted into the chemical structure of propofol, which improves its pharmacological and physicochemical properties and therefore reduces pain during injection [14, 17]. In addition, the lower plasma concentration of ciprofol may also be associated with a lower incidence of injection-site pain relative to propofol [17]. Furthermore, the results of subgroup analysis also prove that injection speed is closely related to pain on injection.

We must admit that our meta-analysis encounters four major limitations. First and foremost, only limited eligible studies with limited sample size were accumulated to evaluate the difference in efficacy and safety between ciprofol and propofol, therefore it was inevitably to compromise the robustness of the merged results. Although inclusion of both RCT and non-RCT may be beneficial for including more eligible studies, we must realize that this strategy will inevitably introduce bias to impair the reliability of findings [43]. Second, this meta-analysis only included studies in which 0.4–0.5 mg/kg ciprofol were used; however, we need to interpret that other doses have also been available for ciprofol, such as 0.2 and 0.3 mg/kg, and all these doses showed promising potential [44, 45]. However, these available doses were not directly compared with propofol, thus resulting in impossibility to evaluate the differences between these doses of ciprofol and propofol. So, future studies need to determine the optimal dose of ciprofol after the advantages of ciprofol compared to propofol has been confirmed. Third, all studies were conducted in China, there was no study conducted in other countries to evaluate the comparative efficacy and safety of ciprofol versus propofol. Therefore, our findings should be interpreted cautiously into other countries. Fourth, publication bias is detected for successful induction rate and the incidence of hypotension, thus inevitably compromising the certainty of the evidence. So, interpretation about these two outcomes should be made with cautious.

## Conclusions

Based on the available data, we conclude that ciprofol may be a promising alternative to propofol for patients undergoing elective surgeries under general anesthesia because of its better anesthesia depth and lower incidence of hypotension and injection-site pain. However, future multicenter studies with large-scale are warranted to validate our findings because only limited eligible studies were cumulated in this meta-analysis.

### Electronic supplementary material

Below is the link to the electronic supplementary material.

Supplementary Material 1

Supplementary Material 2

## Data Availability

All data generated or analysed during this study are included in this published article.

## Abbreviations

CNKI: Chinese national knowledge infrastructure
RCT: Randomized controlled trials
MD: Mean difference
CI: Confidence interval
RR: Risk ratio
GRADE: Grading of recommendations, assessment, development and evaluation
ASA: American society of anesthesiologists
BIS: Bispectral index
MESH: Medical subject heading
SBP: Systolic blood pressure
