# Supplementary material for: Efficacy and safety of ciprofol versus propofol for anesthesia induction in adult patients received elective surgeries: a meta‑analysis
Source: BMC Anesthesiol. 2024 Mar 7;24:93. doi: 10.1186/s12871-024-02479-9 (PMC10919024; doi:10.1186/s12871-024-02479-9)
Supplement: Supplementary file 1 — Supplementary Material 1 [file 12871_2024_2479_MOESM1_ESM.docx]

**Supplementary material**

**
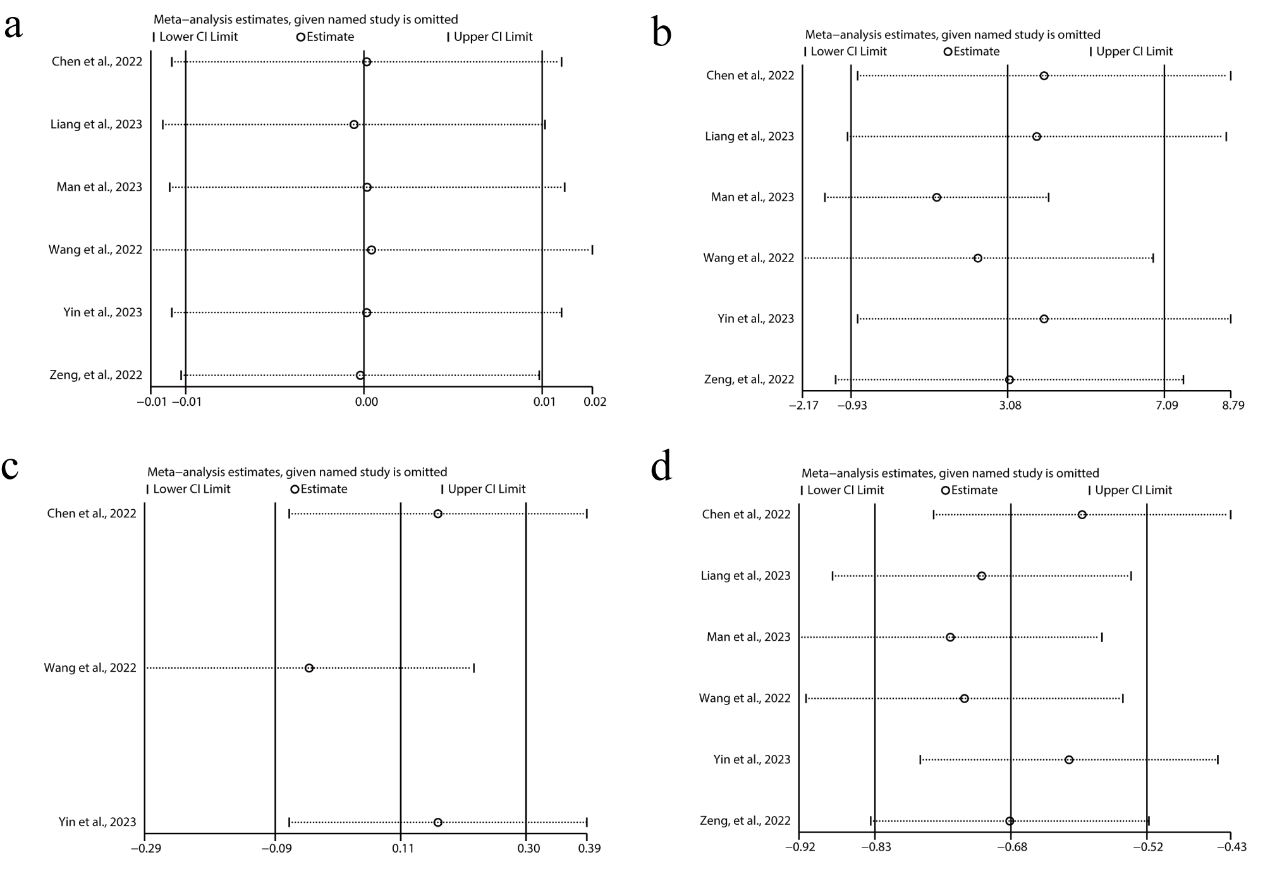
**

**Supplementary figure 1.** Sensitivity analysis of successful induction rate (a), time to onset of successful induction (b), time to disappearance of eyelash reflex (c), and overall estimated mean in BIS between ciprofol and propofol groups.

**
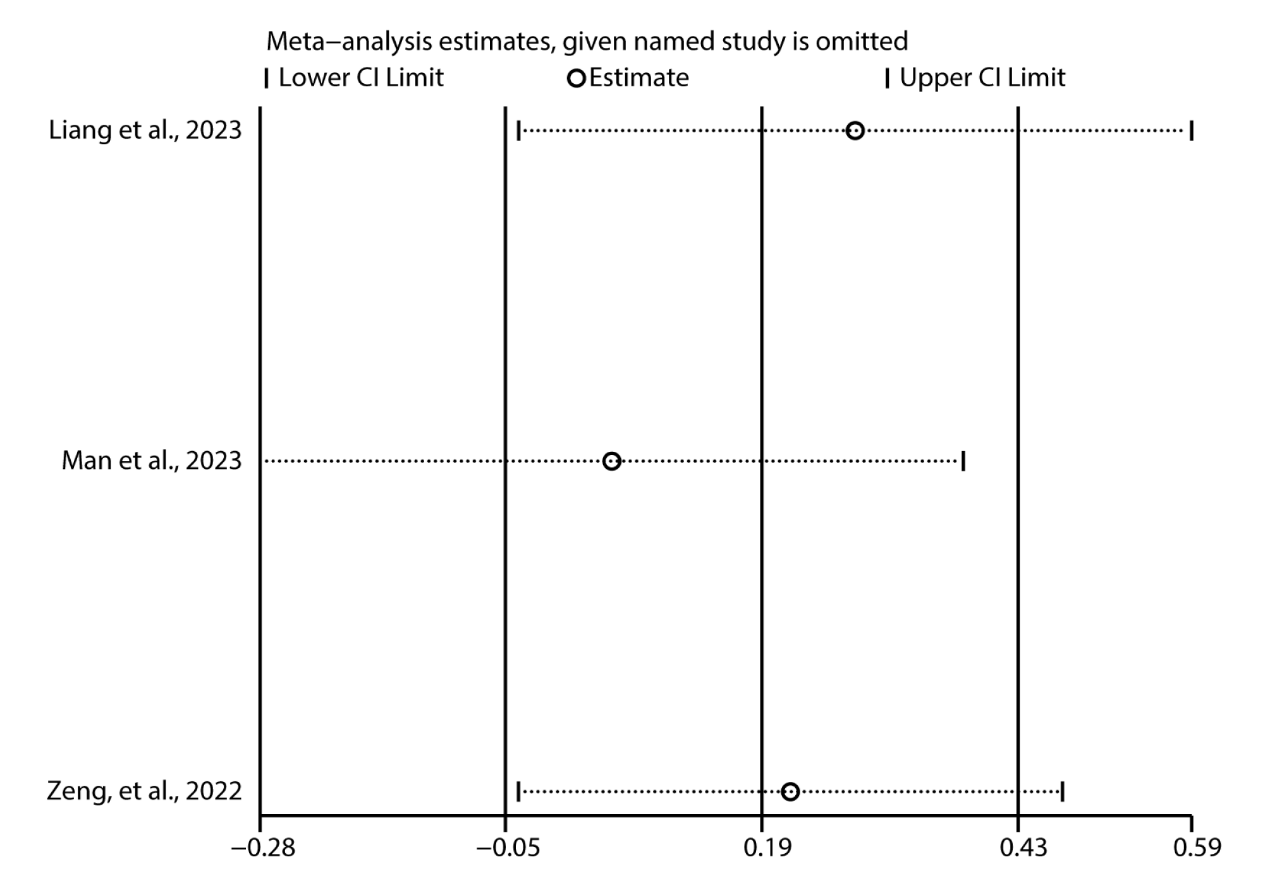
**

**Supplementary figure 2.** Sensitivity analysis of time to full alertness between ciprofol and propofol groups.


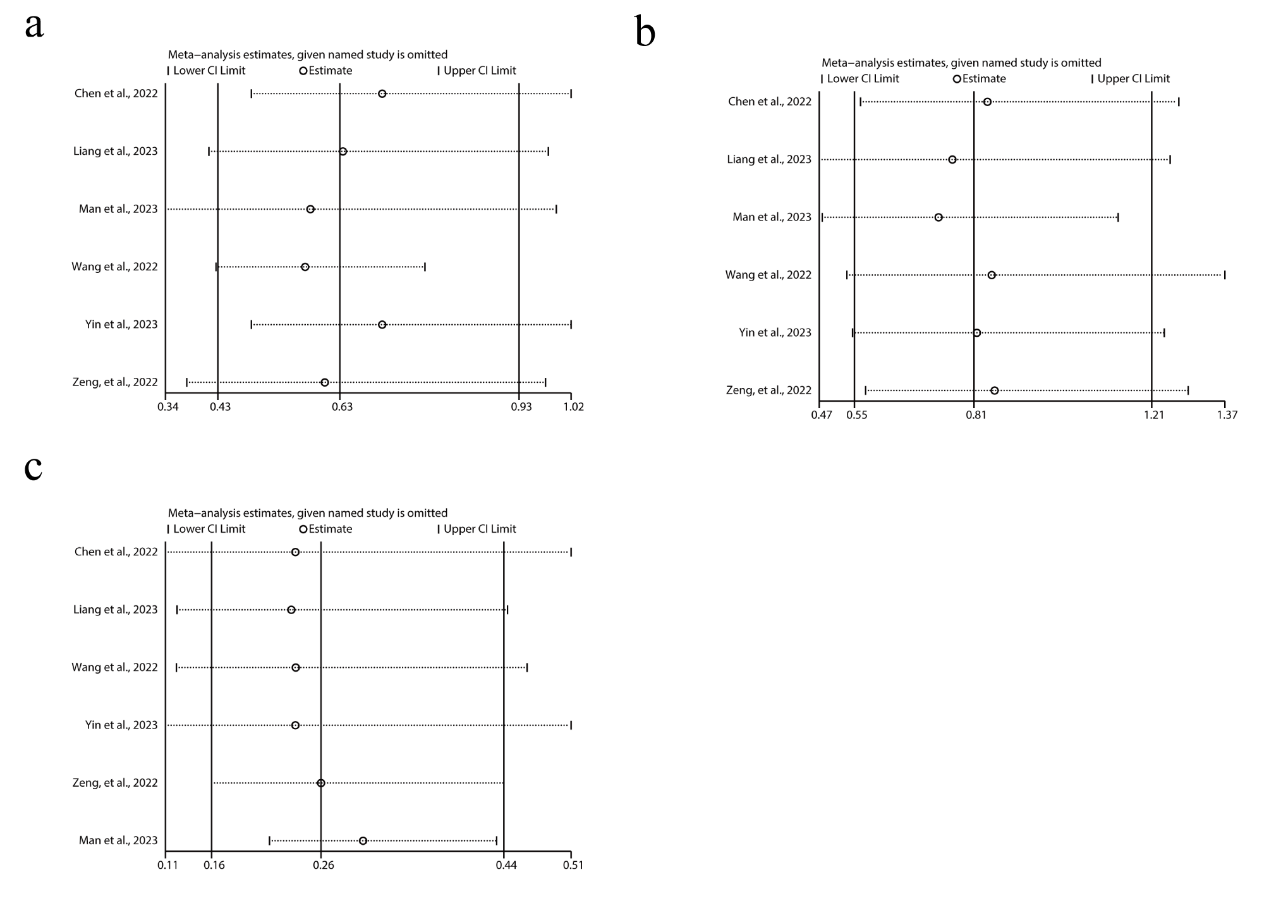


**Supplementary figure 3.** Sensitivity analysis of incidence of hypotension (a), incidence of arrhythmia (b), and incidence of injection-site pain (c) between ciprofol and propofol groups.

**
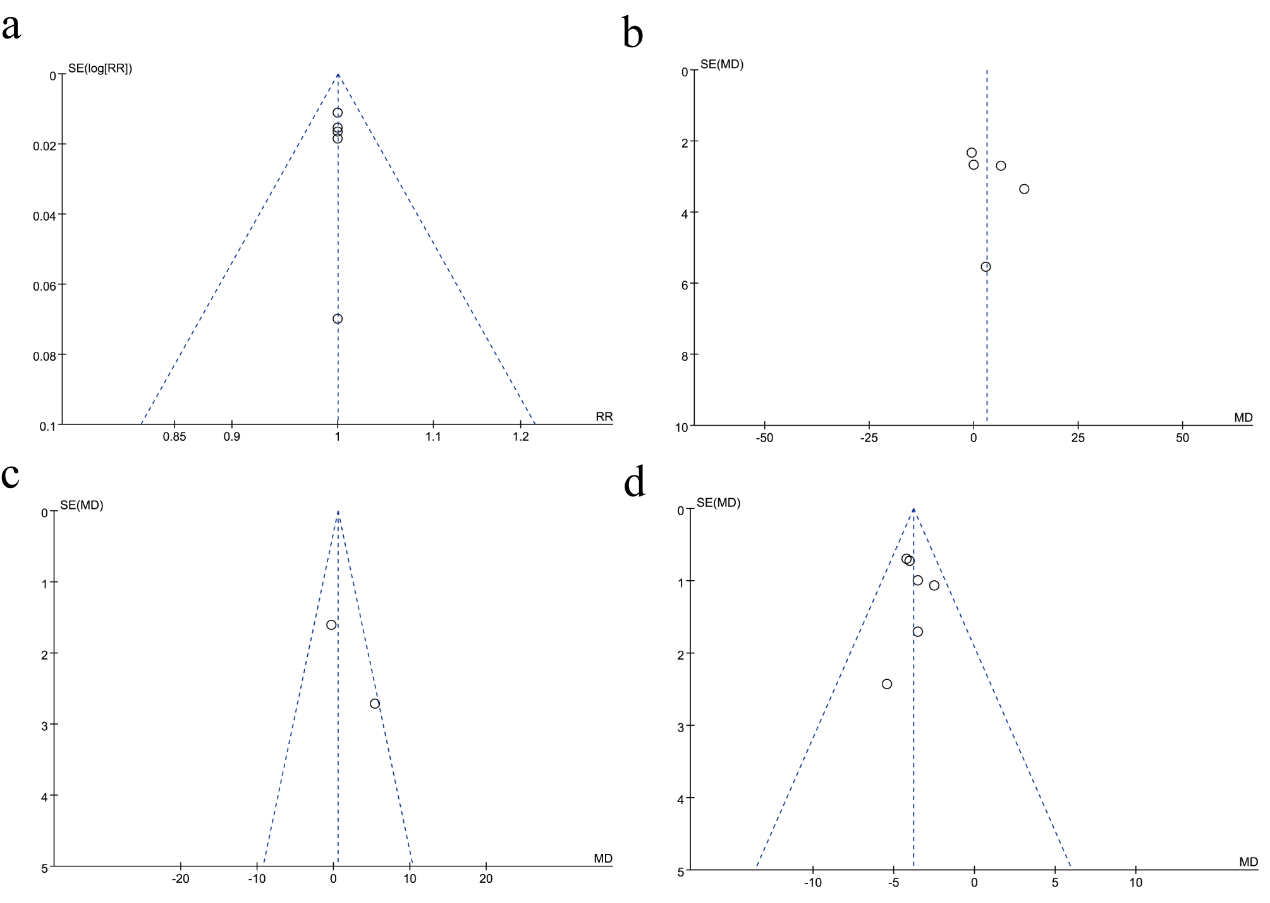
**

**Supplementary figure 4.** Funnel plot of successful induction rate (a), time to onset of successful induction (b), time to disappearance of eyelash reflex (c), and overall estimated mean in BIS between ciprofol and propofol groups.


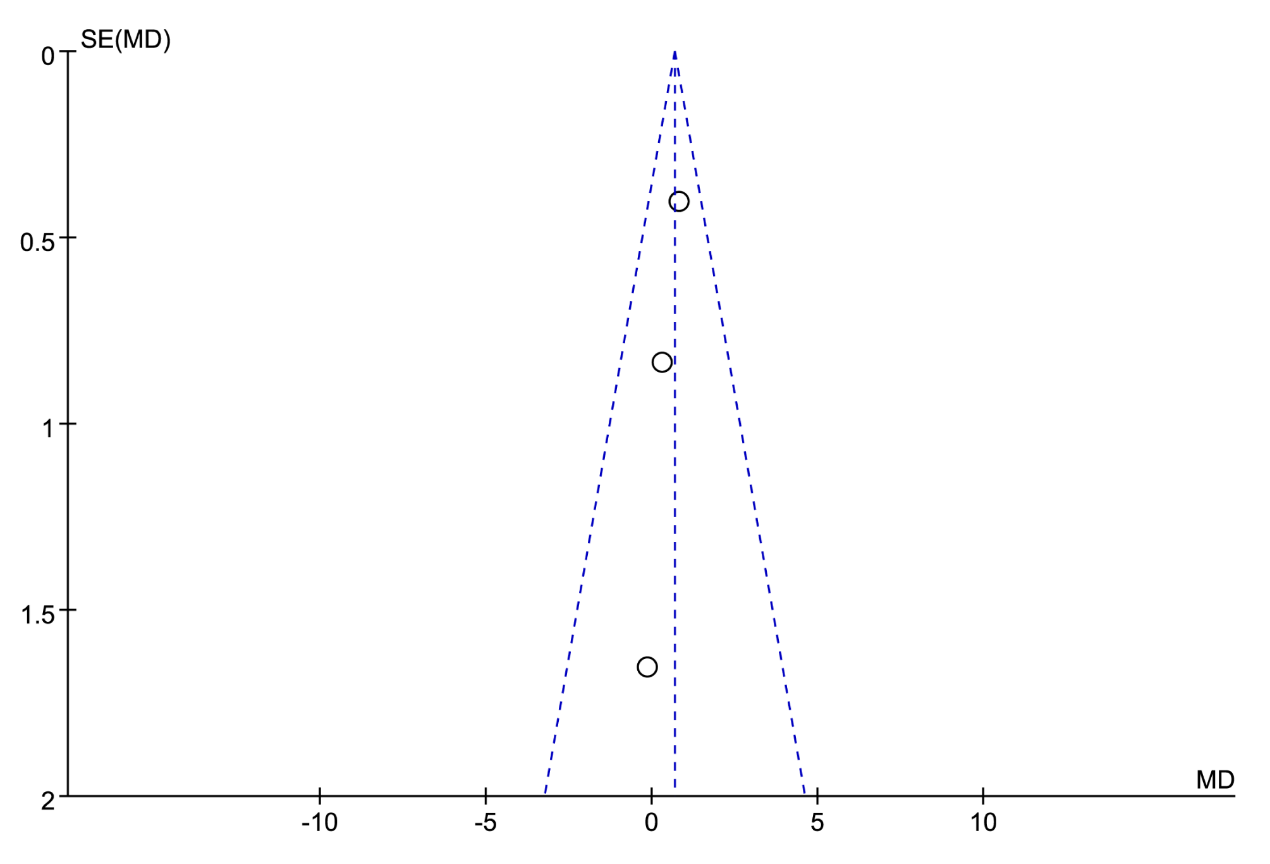


**Supplementary figure 5.** Funnel plot of time to full alertness between ciprofol and propofol groups.

**
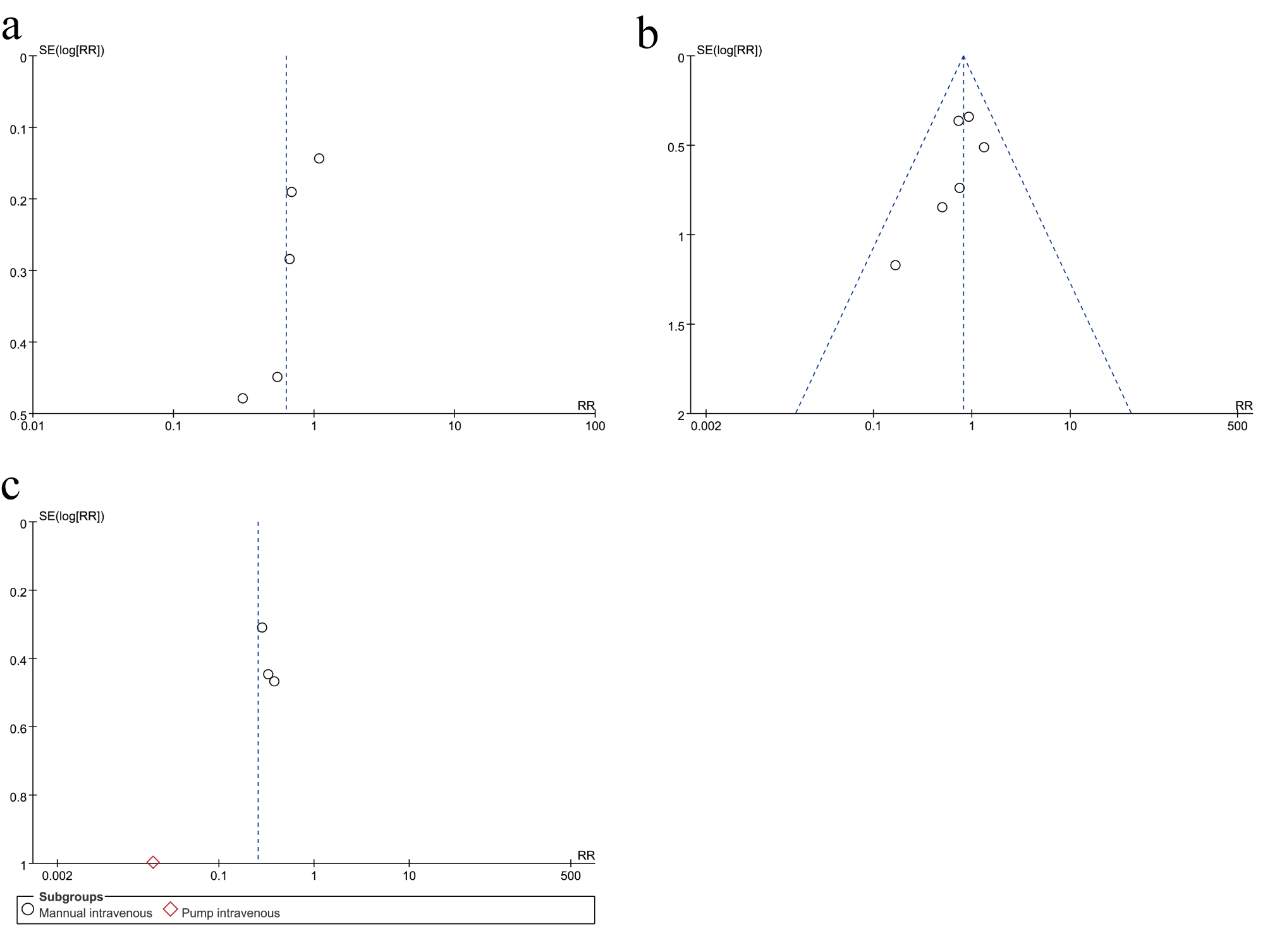
**

**Supplementary figure 6.** Funnel plot of incidence of hypotension (a), incidence of arrhythmia (b), and incidence of injection-site pain (c) between ciprofol and propofol groups.

**Supplementary table 1.** Detailed search strategies of all target databases.

| *Search strategy of PubMed* | | |
| --- | --- | --- |
| No. | Search Details | Results |
| #1 | ((Cipepofol) OR (ciprofol)) OR (HSK3486) | 50 |
| #2 | "Propofol"[Mesh] | 16,869 |
| #3 | ((((((((((Propofol) OR (Disoprofol)) OR (Diprivan)) OR (Disoprivan)) OR (Fresofol)) OR (ICI-35868)) OR (ICI35868)) OR ('ICI 35868')) OR (Ivofol)) OR (Recofol)) OR (Aquafol) | 26,070 |
| #4 | #2 or #3 | 26,070 |
| #5 | (("Randomized Controlled Trial" [Publication Type]) OR "Randomized Controlled Trials as Topic"[Mesh]) OR "Random Allocation"[Mesh] | 850,233 |
| #6 | random* | 1,700,201 |
| #7 | #5 or #6 | 1,701,783 |
| 8 | #1 and #4 and #7 | 24 |

| *Search strategy of Embase* | | |
| --- | --- | --- |
| No. | Search Details | Results |
| #1 | cipepofol OR ciprofol OR hsk3486 | 54 |
| #2 | 'cipepofol'/exp | 48 |
| #3 | #1 OR #2 | 54 |
| #4 | propofol OR disoprofol OR diprivan OR disoprivan OR fresofol OR 'ici 35868' OR ici35868 OR ivofol OR recofol OR aquafol | 73,968 |
| #5 | 'propofol'/exp | 71,383 |
| #6 | #4 OR #5 | 73,968 |
| #7 | random* | 2,245,651 |
| #8 | 'randomized controlled trial'/exp | 787,967 |
| #9 | 'randomized controlled trial (topic)'/exp | 262,295 |
| #10 | 'randomization'/exp | 98,693 |
| #11 | #7 OR #8 OR #9 OR #10 | 2,246,888 |
| #12 | #3 AND #6 AND #11 | 28 |
| #13 | #12 AND [embase]/lim AND 'human'/de | 26 |

| *Search strategy of Cochrane library* | | |
| --- | --- | --- |
| No. | Search Details | Results |
| #1 | (Cipepofol) OR (ciprofol) OR (HSK3486) | 183 |
| #2 | (Propofol) OR (Disoprofol) OR (Diprivan) OR (Disoprivan) OR (Fresofol) | 18,104 |
| #3 | (ICI-35868) OR (ICI35868) OR ('ICI 35868') OR (Ivofol) OR (Recofol) | 20 |
| #4 | (Aquafol) | 9 |
| #5 | #2 or #3 or #4 | 18,104 |
| #6 | MeSH descriptor: [Propofol] explode all trees | 5,695 |
| #7 | #5 or #6 | 18,104 |
| #8 | (random*) | 1,340,565 |
| #9 | MeSH descriptor: [Randomized Controlled Trial] explode all trees | 25,733 |
| #10 | MeSH descriptor: [Randomized Controlled Trials as Topic] explode all trees | 47,401 |
| #11 | MeSH descriptor: [Random Allocation] explode all trees | 23,366 |
| #12 | #8 or #9 or #10 or #11 | 1,340,727 |
| #13 | #1 and #7 and #12 | 89 |

| *Search strategy of Web of Science* | | |
| --- | --- | --- |
| No. | Search Query | Results |
| #1 | Cipepofol (Topic) OR ciprofol (Topic) OR HSK3486 (Topic) | 48 |
| #2 | Propofol (Topic) OR Disoprofol (Topic) OR Diprivan (Topic) OR Disoprivan (Topic) OR Fresofol (Topic) OR ICI-35868 (Topic) OR ICI35868 (Topic) OR 'ICI 35868' (Topic) OR Ivofol (Topic) OR Recofol (Topic) OR Aquafol (Topic) | 32,187 |
| #3 | random* (All Fields) | 2,373,233 |
| #4 | #3 AND #2 AND #1 | 22 |

| *Search strategy of Chinese National Knowledge Infrastructure (CNKI)* | | |
| --- | --- | --- |
| No. | Search Details | Results |
| #1 | (Topic: Cipepofol [precise]) OR (Topic: ciprofol [precise]) OR (Topic: HSK3486 [precise]) | 128 |
| #2 | (Topic: Propofol [precise]) OR (Topic: Disoprofol [precise]) | 36,459 |
| #3 | All fields: random* | 9,561,973 |
| #4 | #1 and #2 and #3 | 43 |
